# Ecophysiological characterization and molecular differentiation of *Culex pipiens* forms (Diptera: Culicidae) in Tunisia

**DOI:** 10.1186/s13071-017-2265-7

**Published:** 2017-07-10

**Authors:** Marwa Beji, Adel Rhim, David Roiz, Ali Bouattour

**Affiliations:** 1 Université Tunis El Manar, Institut Pasteur de Tunis, Laboratoire d’Epidémiologie et de Microbiologie Vétérinaire LR11IPT03, Service d’Entomologie Médicale, 1002 Tunis-Belvédère, Tunisia; 2 Infectious Diseases and Vectors: Ecology, Genetics, Evolution and Control, IRD (Institut de Recherche pour le Développement), Montpellier, France

**Keywords:** *Culex pipiens*, Form *molestus*, Form *pipiens*, Hybrid, Tunisia, Ecology, Autogeny, Microsatellite CQ11, Genetic diversity

## Abstract

**Background:**

The *Culex pipiens* complex (Diptera: Culicidae) includes the most widespread mosquito species in the world. Members of this complex are the primary enzootic and epidemic vectors of the West Nile virus (genus *Flavivirus*) in several countries. The two recognized forms of *Cx*. *pipiens* (Linnaeus, 1758) - *pipiens* and *molestus* *-* exhibit behavioral and physiological differences. Natural populations of *Cx*. *pipiens* were investigated in several sites in Tunisia to evaluate the ecophysiological and molecular characteristics of their forms.

**Results:**

The analysis showed the sympatric presence of *Cx*. *pipiens* forms and hybrids in all studied sites. Of all the tested larvae of *Cx*. *pipiens*, 33.5% were identified as *pipiens*, 30.8% were identified as *molestus*, and 35.6% were identified as hybrids. The *molestus* and hybrid forms were positively correlated with urban habitats and belowground sites while the *pipiens* form was positively correlated with rural habitats and aboveground sites. Autogeny was expressed in all types of habitats and breeding sites. By contrast with the microsatellite CQ11, the two molecular markers, *ace-2* and *cytb*, did not allow differentiation between the *Cx*. *pipiens* forms.

**Conclusions:**

Our study shows the ubiquitous distribution and the plasticity of the different forms of *Cx*. *pipiens* in a wide range of ecological conditions. It suggests that the behavioral traits assigned to the forms of *Cx*. *pipiens* seem to be more flexible than previously assumed. Our analysis also proves that the microsatellite CQ11 remains an efficient tool for distinguishing between *Cx*. *pipiens* forms.

**Electronic supplementary material:**

The online version of this article (doi:10.1186/s13071-017-2265-7) contains supplementary material, which is available to authorized users.

## Background

The epidemic and zoonotic potential of mosquito-borne diseases make mosquitoes an important threat to public health [1]. Mosquitoes of the *Culex pipiens* complex, the most widespread species, are among the principal vectors of diseases including the Rift Valley fever virus (RVFV) and West Nile virus (WNV) [2].

In Tunisia*,* favorable environmental conditions created by rapid urbanization and changing agriculture practices [3, 4] are contributing to the widespread proliferation of *Culex pipiens* mosquitoes and their abundant presence in urban and rural areas. This in turn is leading to the spread of WNV [5, 6], as several recent studies have shown, which has become the most important arboviral disease in Tunisia. WNV is a flavivirus maintained in an enzootic cycle (bird-mosquito-bird transmission), that can lead to encephalitis/meningitis in humans and horses [7]. In Tunisia, three large outbreaks of WNV meningoencephalitis (1997, 2003 and 2012) have led to several deaths [8–11].

The *Cx. pipiens* complex includes six members: *Cx*. *quinquefasciatus* Say, *Cx*. *pipiens pallens* Coquillet, *Cx*. *australicus* Dobrotworsky & Drummond, *Cx*. *globocoxitus* Dobrotworsky and the nominal species, *Cx*. *pipiens* Linnaeus, comprising two forms: *Culex pipiens* f. *pipiens* and *Culex pipiens* f. *molestus* [2, 12]. The difficulty in distinguishing among these forms has made the taxonomy and phylogeny of the *Cx*. *pipiens* complex controversial [13]. Molecular assays have been developed to differentiate the species and forms and to detect hybridization events [14]. Several studies using molecular tools have led to the description of the two forms of *Cx*. *pipiens* in several parts of the world, particularly in North Africa, and have provided evidence of various ecological features. The *pipiens* form is eurygamous (mates in open spaces), anautogenous (requires a blood meal for egg development) and heterodynamic (goes into diapause during the winter). By contrast, the *molestus* form is stenogamous (mates in confined spaces), autogenous (can lay its first batch of eggs without a blood meal) and homodynamic (does not enter diapause) [5, 13, 15–18].

The transmission of WNV is greatly influenced by the ecology, competence, and feeding behavior of the mosquito vectors: *Cx*. *p*. *pipiens* is ornithophilic, feeding mainly on birds, while *Cx*. *p. molestus* is anthropophilic, feeding mainly on mammals, especially humans [19]. Hybrids of the *pipiens* and *molestus* forms have an intermediate host preference that makes them “bridge vectors” for WNV transmission from birds to mammals [18, 19]. The recently reported detection of hybrids of the two forms in several countries presents a complex scenario regarding the hypothesis of a clear behavioral separation among the forms of *Cx*. *pipiens* [20–23].

Taxonomic studies of mosquito vectors, their ecology and their physiology are therefore needed to understand the epidemiology of the diseases that they transmit and to establish surveillance and control programs. Indeed, the unresolved debate about the status of the physiological, ecological and genetic characteristics of the *Cx*. *pipiens* complex makes their ecology, biology and taxonomic status important subjects of study and discussion.

This study used molecular methods to investigate the occurrence and distribution of both forms of *Cx. pipiens* and their hybrids to characterize different populations, to determine their expression and rate of autogeny in different environments in Tunisia. These traits are known to have obvious implications for the vectorial capacity of this mosquito.

## Methods

### Mosquito collection and identification

From 2013 to 2015, mosquito larvae were collected by dipper sampling from 22 sites covering seven bioclimatic zones of Tunisia in both urban and rural habitats and in above- and belowground breeding sites (Table 1). Live larvae were brought to the insectary of the Pasteur Institute of Tunis for identification according to the identification key of Mediterranean Africa mosquitoes [24].

**Table 1:** Characteristics of *Culex pipiens* sampling sites in Tunisia

ID	Bioclimatic zone	Locality	Collection date	Latitude	Longitude	Habitat	Breeding site	No. of specimens analyzed
1	Humid	Cap serrat	August 2015	37°20′23.7″	09°40′11.4″	rural	aboveground	20
2		Skhira	October 2015	37°03′31.0″	09°20′31.0″	urban	aboveground	10
3	Sub-humid	Utique	December 2014	37°04′17.6″	10°00′42.1″	urban	aboveground	17
4		Manar	April 2015	37°01′77.0″	09°52′20.7″	rural	aboveground	20
5		Zaarour	October 2015	37°06′86.8″	09°44′37.8″	rural	aboveground	20
6		Beja oued	December 2013	36°43′88.1″	09°12′31.5″	urban	aboveground	20
7	Higher semi-arid	Cité nozha	June 2015	36°52′00.8″	10°11′78.0″	urban	belowground	20
8		Chotrana	July 2015	36°54′11.1″	10°13′10.0″	urban	aboveground	20
9		Cave 1	October 2015	36°48′10.7″	10°10′45.2″	urban	belowground	20
10		Cave 2	October 2015	36°48′08.6″	10°10′44.4″	urban	belowground	20
11		Cité olympique	September 2015	36°50′36.4″	10°11′69.0″	urban	belowground	20
12		Korba	June 2014	36°34′43.0″	10°51′53.1″	urban	aboveground	20
13		Tastour	May 2015	36°32′41.8″	09°24′16.7″	rural	aboveground	20
14	Middle semi-arid	Cité el Arayes	July 2013	36°24′26.0″	10°08′13.1″	urban	aboveground	20
15	Higher arid	Kairouan	July 2014	35°39′85.1″	10°06′41.9″	urban	aboveground	20
16		Cité bassatin	December 2014	35°10′20.0″	08°49′42.2″	urban	aboveground	20
17		Sidi bouzid	May 2015	34°39′10.0″	09°35′18.3″	urban	aboveground	20
18	Lower arid	Gafsa	December 2014	34°26′43.0″	08°38′15.4″	rural	aboveground	20
19		Route d’el Ain	August 2014	34°44′52.5″	10°45′16.4″	urban	aboveground	20
20		Teboulbou	June 2013	33°50′28.2″	10°07′52.5″	rural	aboveground	20
21	Saharan	Route dghech	April 2015	33°57′21.1″	08°11′04.1″	rural	aboveground	20
22		Douz	December 2013	33°25′90.8″	09°00′95.2″	rural	aboveground	8

A pool of *Cx*. *pipiens* larvae was taken from each site (*n* = 22) and stored in 70% alcohol in preparation for the molecular characterization and genetic analysis of *Cx*. *pipiens* forms. Other larvae pools taken from seven breeding sites representing different combinations of habitat (rural/urban) and breeding site (above/belowground) were reared to adults under laboratory conditions, in order to evaluate their autogenic behavior.

### Molecular identification of *Cx. pipiens* mosquitoes

DNA from individual *Culex pipiens* larvae and adults from each breeding site (Table 1) were extracted using the Cetyltrimethylammonium bromide (CTAB) protocol [25]. Isolated DNA from each sample was stored at -20 °C.

The CQ11 polymorphic microsatellite marker of *Culex pipiens* complex was used to distinguish between form *pipiens* and form *molestus*. The amplification of the CQ11 microsatellite was carried out using sets of primers CQ11F2, molCQ11R and pipCQ11R. The PCR reactions were performed in 20 μl of reaction mix using the cycling conditions listed in Bahnck & Fonseca [26]. Amplified fragments were visualized on a 2% agarose gel. The *pipiens* and *molestus* forms presented a PCR product of 200 bp and 250 bp, respectively. Hybrids exhibited both amplicons (200 bp/250 bp) [26].

A second PCR was subsequently used to detect polymorphism in the nucleotide sequence of the *ace-2* gene of the different forms of *Cx*. *pipiens* and to test its usefulness as a nuclear marker for form identification. Sequences of sections of exons 2 and 3 and the entire intron 2 in the *ace-2* gene (the ACE locus) were obtained using the oligonucleotide primers, specific for *Cx*. *pipiens* (*s.s*.), F1457 and B1246 as described by Bourguet et al. [27]. PCR products were run on a 1.5% agarose gel and showed a band of 714 bp specific of *Cx. pipiens*.

In addition, samples were analyzed by PCR targeting the *cytb* gene that was used in species identification [28–30] to detect any polymorphism in the nucleotide sequence of *Cx*. *pipiens* forms. Amplification of the *cytb* gene was carried out using the primers cytb-F and cytb-R [30]. Polymerase chain reaction products were run on a 1% agarose gel and displayed a band of 853 bp specific of *Cx*. *pipiens*.

### Sequencing

Some PCR products obtained by targeting the CQ11, *ace-2* and *cytb* were randomly chosen and sequenced to confirm the PCR results and to determine whether nucleotide polymorphisms were informative to distinguish between *Cx*. *pipiens* forms. PCR products were purified using the ExoSAP cleanup procedure (Amersham Biosciences, Piscataway, NJ, USA). Cycle sequencing was performed using BigDye Terminator v.3.1 Cycle Sequencing Kit (Applied Biosystems, Foster City, CA, USA) and analyzed using a capillary automated sequencer 3500 Genetic Analyzer (Ruo. Hitachi, Foster City, CA, USA). Sequences were aligned using BioEdit 7.1.9 [31] and identified by comparison with sequences deposited in the GenBank database.

### Determination of autogeny

To evaluate the expression of autogeny according to the type of habitat and breeding site, *Cx*. *pipiens* larvae from ID3, 4, 6, 9, 11 and 13 sites (Table 1) were raised in the insectary under controlled conditions (25 ± 2 °C; 70 ± 10% relative humidity, and a 12:12 h light:dark photocycle). Larvae were fed fish flakes and brewer’s yeast. Emerging males and females of *Cx*. *pipiens* housed in cages (20 × 20 × 20 cm) were given access to a cotton pad soaked in a 10% sugar solution and an oviposition small tray containing deionized water that was inspected daily for 30 days for the presence of egg-rafts. We subsequently calculated the number of fertile egg-rafts (which produce larvae) to estimate the percentage of autogenous females.

In a second test evaluating the expression of autogeny by form of *Cx*. *pipiens*, two types of breeding sites (aboveground ID8 and belowground ID11) were chosen. Pupae were separated individually in glass tubes of distilled water until adults emerged. The adults were isolated by couples (one male and one female) in cups covered with a mesh screen with access to a honey solution and an oviposition tray. The presence of egg-rafts was recorded daily for 30 days. During this time, females that laid eggs without blood-feeding were considered to be autogenous. This test was replicated by visiting the two sites three times (once a month). We started our experiment with 60 couples from ID8 and 57 from ID11 but we used molecular analysis only for the survived females to determine the form.

### Data analysis

The relationship between the form of *Cx*. *pipiens* and bioclimatic area, breeding site, habitat and autogenic behavior was analyzed using a Generalized Linear Model (GLM) with Poisson distribution (as the data were overdispersed). Statistical analyses and figures were carried out in R 3.2.2.

### Nucleotide sequence accession numbers

Sequence data were deposited in the GenBank database under the accession numbers KY744191–KY744222.

## Results

During our study, 1517 mosquito larvae were collected from 22 sites in Tunisia’s seven bioclimatic zones (Table 1) and identified as *Cx*. *pipiens* (*n* = 989), *Cx*. *theileri* (*n* = 404), *Cx*. *perexiguus* (*n* = 11), *Cx*. *impudicus* (*n* = 9), *Ochlerotatus caspius* (*n* = 16), *O. detritus* (*n* = 10), *Anopheles labranchiae* (*n* = 28), *Culesita longiareolata* (*n* = 48), *Orthopodomyia pulchripalpis* (*n* = 1) and *Uranotaenia unguiculata* (*n* = 1).

Among the collected larvae, 415 larvae of *Cx*. *pipiens* were molecularly typed using CQ11, *ace-2* and *cytb* PCR at the form level. Furthermore, approximately 574 larvae were raised to obtain adults to determine their expression of autogeny.

### Occurrence and distribution of *Cx*. *pipiens* forms

**Fig. 1:**
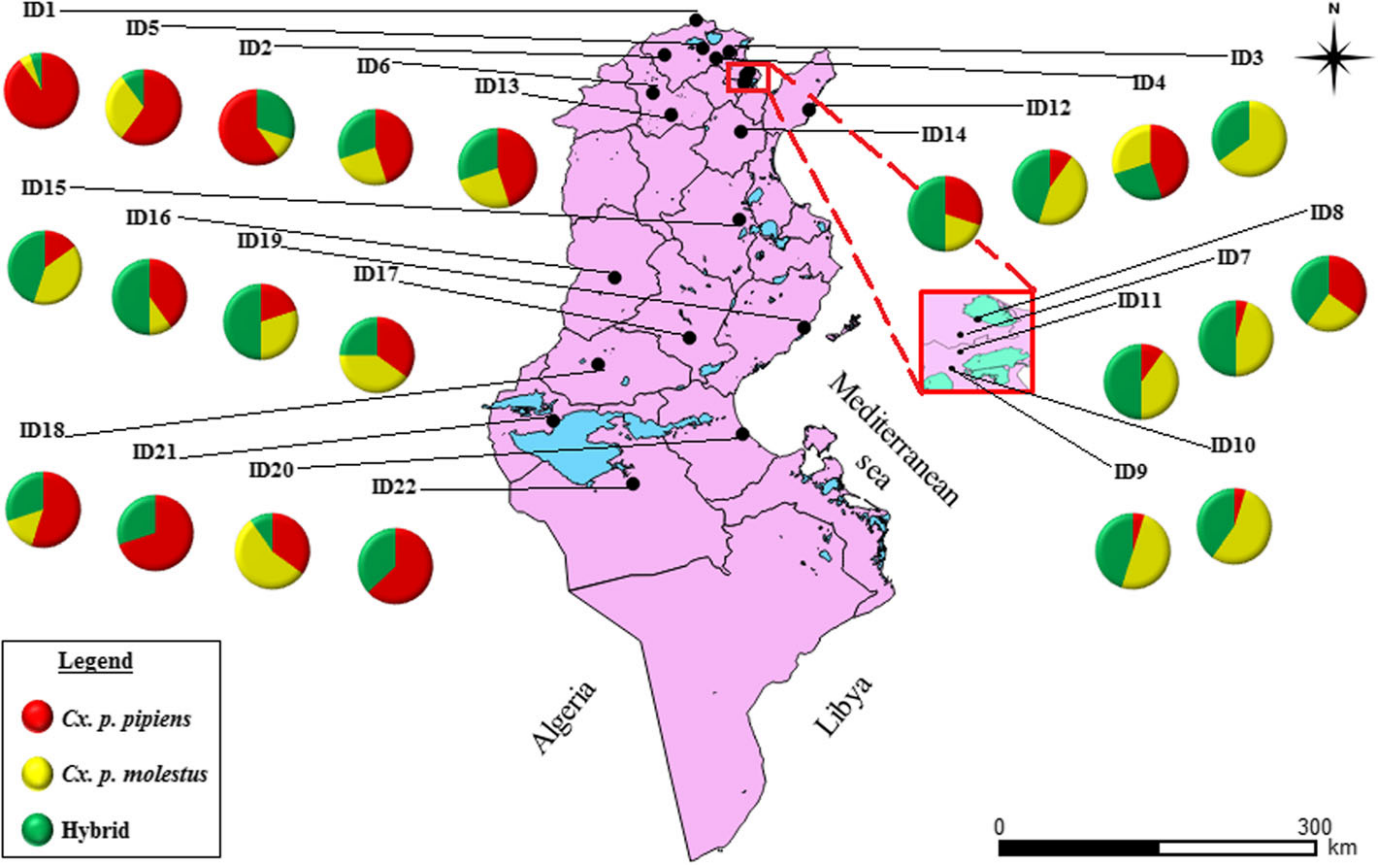
Distribution of *Culex pipiens* forms. Composition of the *Culex pipiens* biotypes of 22 field-collected populations in Tunisia using the CQ11 assay

Amplification of the CQ11 microsatellite showed different frequencies of the *Cx*. *pipiens* forms in all 22 sites (Fig. 1). Of the 415 larvae that were analyzed, 139 (33.50%) specimens were *pipiens* form, 128 (30.84%) were *molestus* form, and 148 (35.66%) were hybrids (Additional file 1: Table S1). A statistical analysis (using GLM with Poisson distribution) showed no significant differences in the frequencies of forms according to bioclimatic zones (Additional file 2: Table S2). Of the 22 sites, 19 (86.36%) were characterized by a sympatric presence of the two *Cx*. *pipiens* forms with their hybrids; two sites [ID21 and ID22; 2/22 (9.1%)] shared *pipiens* form and hybrids, and one site [ID3; 1/22 (4.55%)] shared *molestus* form and hybrids. No pure sites (only *pipiens* or *molestus*) were observed.

**Fig. 2:**
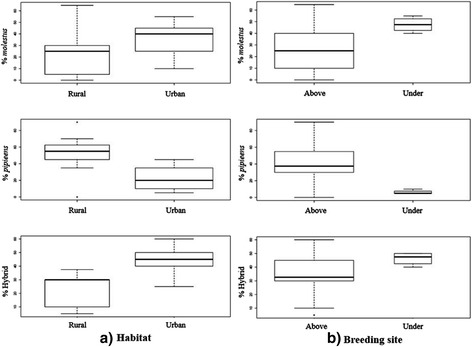
Boxplot showing the percentage of *Cx*. *pipiens* forms according to the type of habitat (**a**) and the type of breeding site (**b**)

Regarding habitat type (Fig. 2a), statistical analysis showed that the frequency of *Cx*. *pipiens* f. *pipiens* was significantly higher in rural locations than in urban locations; that *Cx*. *pipiens* f. *molestus* was significantly more abundant in urban areas than in rural areas and that the frequency of hybrids was significantly higher in urban sites than in rural sites (see Additional file 3: Table S3).

Statistical analysis also showed that the proportion of the *molestus* form was significantly higher in belowground breeding sites (see Additional file 3: Table S3; Fig. 2b) whereas a higher rate of *pipiens* form was observed in aboveground sites and hybrids were significantly more frequent in belowground sites than in aboveground sites (see Additional file 3: Table S3).

### Sequencing and genetic analyses

To clarify the taxonomic status of the *Cx*. *pipiens* forms determined by PCR, we sequenced 12 randomly chosen amplicons obtained by targeting CQ11, *ace-2* and *cytb* genes. The results allowed us to compare three available molecular methods to distinguish the *Cx*. *pipiens* forms.

#### CQ11 microsatellite variability

Eight PCR products of *pipiens* (*n* = 4) and *molestus* (*n* = 4) forms were sequenced (GenBank: KY744215–KY744222). A BLAST analysis of these sequences confirmed the results of the PCR but revealed some variability among available sequences in GenBank. The four sequences of *pipiens* form (GenBank: KY744215–KY744218) showed significant similarity (98–99%) with sequences of *Cx*. *p*. *pipiens* described in the UK and the four sequences of *molestus* form (GenBank: KY744219–KY744222) showed significant similarity (99–100%) with sequences of *Cx*. *p. molestus* described in the UK (Table 2).

**Table 2:** Comparative molecular identification of *Cx. pipiens* forms

ID^a^	CQ11 PCR	CQ11 sequences				*ace-2* sequences				*cytb* sequences			
		Form	GenBank ID	Reference sequence	Similarity (%)	Form	GenBank ID	Reference sequence	Similarity (%)	Form	GenBank ID	Reference sequence	Similarity (%)
19	P	P	KY744215	DQ470145.1	99	*Cx*. *pipiens*	KY744203	AY196910.1	99	*Cx*. *pipiens*	KY744191	HQ724614.1	100
13	P	P	KY744216	DQ470145.1	99	*Cx*. *pipiens*	KY744204	AY196910.1	99	*Cx*. *pipiens*	KY744192	HQ724614.1	100
5	P	P	KY744217	DQ470148.1	98	*Cx*. *pipiens*	KY744205	AY196910.1	100	*Cx*. *pipiens*	KY744193	HQ724616.1	100
												HQ724614.1	99
16	P	P	KY744218	DQ470142.1	99	*Cx*. *pipiens*	KY744206	AY196910.1	100	*Cx*. *pipiens*	KY744194	HQ724616.1	100
												HQ724614.1	99
13	M	M	KY744219	DQ470150.1	100	*Cx*. *pipiens*	KY744207	AY196910.1	99	*Cx*. *pipiens*	KY744195	HQ724614.1	100
15	M	M	KY744220	DQ470150.1	100	*Cx*. *pipiens*	KY744208	AY196910.1	99	*Cx*. *pipiens*	KY744196	HQ724614.1	100
3	M	M	KY744221	DQ470149.1	99	*Cx*. *pipiens*	KY744209	AY196910.1	99	*Cx*. *pipiens*	KY744197	HQ724614.1	100
5	M	M	KY744222	DQ470149.1	99	*Cx*. *pipiens*	KY744210	AY196910.1	100	*Cx*. *pipiens*	KY744198	HQ724616.1	100
												HQ724614.1	99
15	H	–		–	–	*Cx*. *pipiens*	KY744211	AY196910.1	99	*Cx*. *pipiens*	KY744199	HQ724614.1	100
21	H	–		–	–	*Cx*. *pipiens*	KY744212	AY196910.1	99	*Cx*. *pipiens*	KY744200	HQ724614.1	100
7	H	–		–	–	*Cx*. *pipiens*	KY744213	AY196910.1	99	*Cx*. *pipiens*	KY744201	HQ724614.1	100
13	H	–		–	–	*Cx*. *pipiens*	KY744214	AY196910.1	99	*Cx*. *pipiens*	KY744202	HQ724616.1	100
												HQ724614.1	99

*Abbreviations*: P, *Cx. pipiens* f. *pipiens*; M, *Cx. pipiens *f. *molestus*; H, hybrid

^a^Details in Table 1

#### *Ace-2* gene variability

The DNA of larvae samples including those previously sequenced for CQ11 locus (*n* = 4 *pipiens*; *n* = 4 *molestus*) and hybrid samples (*n* = 4), were amplified and sequenced targeting the *ace-2* gene (714 bp) (GenBank: KY744203–KY744214). A BLAST analysis of these sequences (*n* = 12) showed a 99–100% similarity with a sequence of *Cx*. *pipiens* previously described in the USA (AY196910.1) [32].

Multiple alignments of our sequences (*n* = 12) showed that variable sites were mainly in intron 2 (non-coding region from 118 bp to 477 bp), which is characterized by a higher mutation rate [33].

#### *Cytb* gene variability

The same DNA samples (*n* = 12) previously sequenced for the nuclear gene (*ace-2*) were amplified and sequenced for the mitochondrial gene (*cytb*) (GenBank: KY744191–KY744202).

Following the BLAST analysis, 4 of the 12 analyzed DNA sequences were 100% identical to the sequence of *Cx*. *p*. *pipiens* from Turkey and shared a 99% similarity with *Cx*. *p*. *pipiens* previously described in Tunisia (Table 2). The remaining 8 sequences were 100% similar to the sequence of *Cx*. *p*. *pipiens* from Tunisia available on GenBank.

Multiple alignments of sequences showed no variability among *Cx*. *pipiens* forms as identified by the CQ11 microsatellite.

### Autogeny

**Fig. 3:**
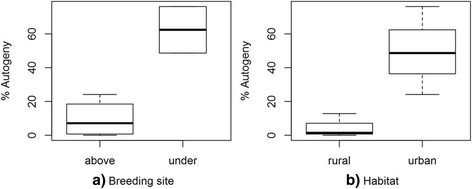
Boxplot showing the percentage of autogeny according to the type of the breeding site (**a**) and the type of habitat (**b**)

To determine the autogenic expression of the field-collected mosquitoes, adults (males and females) from six breeding sites (ID3, 4, 6, 9, 11 and 13) were reared in six cages in the insectary. Females that produced fertile eggs without access to a blood meal were considered autogenous. The results of this test are represented in Fig. 3 and Additional file 4: Table S4. Statistical analysis shows that the highest proportion of autogenous mosquitoes were found in belowground breeding sites (Fig. 3a; Additional file 5: Table S5) and in urban habitats (Fig. 3b; Additional file 5: Table S5).

In a second test, we evaluated the *Cx*. *pipiens* form versus autogeny in two types of breeding sites (ID8: aboveground; ID11: belowground) by placing couples from each site in cups and following them for 30 days for the presence of egg-rafts. These two sites were visited three times to replicate the test. From 117 tested couples (60 couples for ID8 and 57 for ID11), survived females (*n* = 90) were subsequently identified molecularly at the form level targeting the CQ11 microsatellite.

**Fig. 4:**
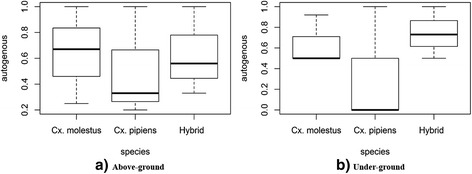
Boxplot showing the percentage of autogeny of the *Cx*. *pipiens* forms according to the type of the breeding site: aboveground (**a**) and belowground (**b**)

The CQ11 assay of autogenous females collected from ID8 (aboveground) showed that 50% (11/22) of the samples were *Cx*. *p. molestus*, 36.36% (8/22) were hybrids, and 13.64% (3/41) were *Cx*. *p*. *pipiens*. From the belowground ID11 site, 52.78% (19/36) of the samples belonged to the *molestus* form, 44.44% (16/36) were hybrids and the remaining 2.78% (1/36) corresponded to the *pipiens* form (Additional file 6: Table S6; Fig. 4).

Anautogenous females from the ID8 site were 42.11% (8/19) hybrids, 31.58% (6/19) *Cx*. *p*. *pipiens* and 26.32% (5/19) *Cx*. *p. molestus*. From the ID11 site, 61.54% (8/13) of anautogenous females were the *molestus* form, 38.46% (5/13) hybrids and 0% were the *pipiens* form (Additional file 6: Table S6; Fig. 4).

Statistical analyses showed that *Cx*. *p. molestus* was the most autogenous form in the two types of breeding sites (50% in ID8; 52.78% in ID11) and that autogeny was negatively related to the *pipiens* form*.* Statistical analyses also demonstrated that differences between *Cx*. *p. molestus* and hybrids concerning the rate of autogeny in the aboveground and belowground site were not significant (Additional file 7: Table S7).

## Discussion

Of the 1517 mosquito larvae collected from 22 breeding sites distributed in seven different climatic zones of Tunisia, *Culex pipiens* was the most abundant (65%). This mosquito species occurs throughout temperate latitudes and is involved in the transmission of West Nile virus in Tunisia [5, 34].

In this study, we investigated the physiological, ecological, and genetic characteristics of the *Cx*. *pipiens* populations that we collected. The screening of 415 *Cx*. *pipiens* larvae by CQ11 microsatellite showed the presence of two *Cx*. *pipiens* forms (*pipiens* and *molestus*) and their hybrids. All 22 breeding sites contained both *Cx*. *pipiens* forms and hybrids with varying frequencies. A previous study in Tunisia has already identified *pipiens* and *molestus* and their hybrids occurring in sympatry in different aboveground collection sites, but found no *pipiens* form in belowground sites [16]. Previous studies had shown that the different forms of *Cx*. *pipiens* were separated primarily on the basis of their ecological and physiological characteristics and that they occupied distinct habitats [35–38]. By contrast, our results showed the co-occurrence of both *Cx*. *pipiens* forms and their hybrids in different breeding sites, matching other studies conducted in Algeria [39, 40], Morocco [5], several European countries, i.e. Portugal [21, 41], the Netherlands [22] and Italy [23], and in the USA [20]. Whereas the *molestus* form was previously considered to be strictly anthropophilic and limited to belowground and confined breeding sites, we found that it can occur naturally in open and aboveground habitats. Similar observations were reported in other studies in Chicago and New York (USA) and in Algeria [39, 42].

The sympatric occurrence thus favors mating between the two forms and the emergence of hybrid populations. Indeed, hybrids were found in all breeding sites shared by the two parental forms. Interestingly, our results revealed that hybrids share the same ecological preferences of the *molestus* form, which may have increased the transmission of WNV to humans. The significant role played by hybrids in transmitting pathogens is well established; their opportunistic feeding behavior acts as a bridge vector for WNV transmission between birds and humans [4, 19, 20, 43, 44].

These findings confirm that *Cx*. *pipiens* forms can share the same site regardless of breeding site or habitat, without competitive exclusion. They also point to the adaptive capacity of *Cx*. *pipiens* forms to various environments and support the species’ ecological and physiological adaptability to urbanization [4, 45]. Man-made artificial habitats including canals, storage lakes, swimming pools, gardens and stormwater drainage systems, act as new breeding sites that primarily favor *Cx*. *pipiens*. Changes in climate may also influence mosquito physiology and ecology. Rises in temperature are known to influence adult flight activity, the digestion of blood meals, and egg development [46, 47]. Indeed, exposure to high temperatures can cause genetic mutations such as DNA methylation, which seems to play a role in facilitating plasticity in response to environmental stress [48, 49].

Insofar as the CQ11 microsatellite may overestimate the rate of hybrids when compared with full microsatellite analysis [42], we chose to compare the CQ11 amplification and sequencing results with the *ace-2* and *cytb* genes to evaluate their utility for discriminating *Cx. pipiens* forms.

The sequencing of the CQ11 PCR product confirmed the presence of the *pipiens* and *molestus* forms in the sites studied, and confirms the results of other, similar studies. It constitutes a valuable tool for characterizing the *Cx*. *pipiens* forms in Tunisia and remains the most appropriate tool of confirmation, especially given the evolved ecological differences.

The amplification and sequencing of the PCR products targeting the *ace-2* and *cytb* did not show any specific differences in sequences and did not allow the recognition of the different forms. Even though, when comparing two available sequences of *ace-2* gene in GenBank [from Iran (*pipiens*) and from Japan (*molestus*)], the result did show differences in two nucleotide positions (Additional file 8: Table S8). In fact, our results showed that the two forms of *Cx*. *pipiens* are genetically too close to permit their discrimination using a nuclear (*ace-2*) [32] or mitochondrial (*cytb*) genes. Indeed, previous research comparing different mitochondrial genes (*cox*1, *nad*4 and *12S*) confirmed their limited utility for the intraspecific differentiation of *Cx*. *pipiens* [50]. Thus, to date the molecular analyses seeking to differentiate the forms of *Cx*. *pipiens* indicate that the CQ11 locus remains the most promising diagnostic marker [21, 41] as it makes it possible to differentiate the two forms of *Cx*. *pipiens* and their hybrids.

This study shows the simultaneous occurrence of the two forms of *Cx*. *pipiens* with their hybrids in the same breeding sites. It is still necessary to determine whether they are also autogenous, a character always related to the *molestus* form that occur in urban belowground sites [13]. Our results demonstrated that autogeny was expressed in the collected females from above- and belowground sites, but that it was significantly higher in the latter. This could be due to the fact that subterranean mosquitoes adapt to habitats where potential blood meals are scarce by developing autogeny [51]. This suggests that *Cx*. *pipiens* has a capacity to adapt to the absence of nutrition by carrying over reserves from the larval stage to produce eggs. In aboveground sites, the low percentage of autogeny in tested females corroborated studies conducted in North Africa [16, 52], East Asia [53] and Portugal [54].

Autogeny was expressed more in urban than in rural habitats, suggesting that environmental factors such as limited access to a breeding site, larval nutrition and photoperiod, would affect it. Its expression may also be influenced by the non-availability of hosts for a blood meal and limited space for mating [35]. This high expression of autogeny may be related to the high proportion of *molestus* form observed in this habitat, which supports previous studies conducted in Australia and Italy [23, 51]. Our findings also demonstrate that a low proportion of *pipiens* form can also lay eggs without blood meals, a rare observation that corroborates a study in Portugal [21] and further confirms the ecological and physiological flexibility of the *Cx*. *pipiens* mosquito. We also observed that some *molestus* females can be anautogenous. Poor adaptation to insectary conditions may cause gonotrophic dissociation, which could explain the absence of oviposition in families that might otherwise be autogenous [21].

## Conclusions

Our study shows the ubiquitous distribution of *Cx*. *pipiens* in Tunisia and provides evidence for the sympatric occurrence of *Cx*. *pipiens molestus*, *Cx*. *pipiens pipiens* and their hybrids. We also demonstrated the great plasticity of this complex of mosquitoes to a wide range of ecological conditions. The results suggest that the behavioral traits assigned to the forms of *Cx*. *pipiens* seem to be more flexible than previously assumed, especially the dispersion of *molestus* and hybrids forms. Our observations also highlight the abundance of autogeny, which is expressed in *molestus* and hybrids in belowground and aboveground sites. Our analysis proved that CQ11 microsatellite continues to be an appropriate molecular tool for the identification of the *Cx*. *pipiens* forms and their hybrids. However, further studies are needed to develop additional molecular markers given the genetic complexity of *Cx*. *pipiens* and the limitation of the use of a single molecular marker.

## Additional files

Additional file 1: Table S1. Frequencies of *Cx. pipiens* forms determined by PCR targeting the CQ11 microsatellite. (PDF 89 kb)

Additional file 2: Table S2. Relationship between the bioclimatic region and the proportion of *Cx. pipiens* forms based on a Generalized Linear Model (GLM) with Poisson distribution. (PDF 98 kb)

Additional file 3: Table S3. Relationship between the different types of habitat and breeding site and the proportion of *Cx. pipiens* based on a Generalized Linear Model (GLM) with Poisson distribution. (PDF 98 kb)

Additional file 4: Table S4. Autogenic expression in field female mosquitoes. (PDF 168 kb)

Additional file 5: Table S5. Results of the relationship between habitat type and breeding site type and the percentage of autogeny of *Cx. pipiens* mosquitoes, based on a Generalized Linear Model with Poisson distribution. (PDF 88 kb)

Additional file 6: Table S6. Autogeny according to *Cx. pipiens* form. (PDF 89 kb)

Additional file 7: Table S7. Results of the relationship between percentage of autogeny of *Cx. pipiens* forms and the type of breeding site, based on a Generalized Linear Model with Poisson distribution. (PDF 313 kb)

Additional file 8: Table S8. Nucleotide variants in 714 bp of the acetylcholine esterase 2 gene in *Cx. pipiens* (PDF 160 kb)

## Abbreviations

*Ace*: Acetylcholinesterase
CTAB: Cetyltrimethylammonium bromide
*cytb*: cytochrome b
Exo: Exonucléase I
GLM: Generalized linear model
ID: Identification number
RVFV: Rift Valley fever virus
SAP: Shrimp alcaline phosphatase
WNV: West Nile virus
