# Supplementary material for: Ecophysiological characterization and molecular differentiation of Culex pipiens forms (Diptera: Culicidae) in Tunisia
Source: Parasit Vectors. 2017 Jul 10;10:327. doi: 10.1186/s13071-017-2265-7 (PMC5504560; doi:10.1186/s13071-017-2265-7)
Supplement: Supplementary file 1 — Frequencies of Cx. pipiens forms determined by PCR targeting the CQ11 microsatellite. (PDF 89 kb) [file 13071_2017_2265_MOESM1_ESM.pdf]

Table S1. Frequencies of *Cx. pipiens* forms determined by PCR targeting the CQ11 microsatellite.

			**Habitat**		**Breeding site**	
**Biotype**	N	%	Rural (%)	Urban (%)	Above (%)	Under (%)
pipiens	139	33.50	85 (61.15)	54 (38.85)	134 (96.40)	5 (3.60)
molestus	128	30.84	42 (32.81)	86 (67.19)	90 (70.31)	38 (29.69)
**hybrid**	148	35.66	38 (25.68)	110 (74.32)	111 (75)	37 (25)
**Total**	415	100	165	250	335	80

Description of data

This file details the distribution of each form of *Cx. pipiens* in two types of a habitat (rural and urban) and in two type of breeding site (above-ground and under-ground) in Tunisia. The differentiation between forms was determined by PCR targeting the CQ11 microsatellite.
