# Supplementary material for: Ecophysiological characterization and molecular differentiation of Culex pipiens forms (Diptera: Culicidae) in Tunisia
Source: Parasit Vectors. 2017 Jul 10;10:327. doi: 10.1186/s13071-017-2265-7 (PMC5504560; doi:10.1186/s13071-017-2265-7)
Supplement: Supplementary file 2 — Relationship between the bioclimatic region and the proportion of Cx. pipiens forms based on a Generalized Linear Model (GLM) with Poisson distribution. (PDF 98 kb) [file 13071_2017_2265_MOESM2_ESM.pdf]

Table S2. Relationship between the bioclimatic region and the proportion of *Cx. pipiens* forms based on a Generalized Linear Model (GLM) with Poisson distribution.

Dependent variable	*Independent variable*		*Estimate*	*Standard error*	*Z value*	*P (> |Z|)*
Humid	Species	Intercept	-2.590	2.582	-1.003	0.316
		% *Cx.pip.pip*	2.079	2.739	0.759	0.448
		% Hybrid	1.466	2.864	0.512	0.609
Sub-humid	Species	Intercept	-1.0147	0.8305	-1.222	0.222
		% *Cx.pip.pip*	0.0339	1.1646	0.029	0.977
		% Hybrid	-0.3228	1.2814	-0.252	0.801
Higher semi-arid	Species	Intercept	-0.89859	0.59235	-1.517	0.129
		% *Cx.pip.pip*	-0.90756	1.10474	-0.822	0.411
		% Hybrid	0.05129	0.82717	0.062	0.951
Middle semi-arid	Species	Intercept	-1.6094	2.2361	-0.720	0.472
		% *Cx.pip.pip*	0.4055	2.8867	0.140	0.888
		% Hybrid	0.9163	2.6457	0.346	0.729
Higher arid	Species	Intercept	-1.204e+00	1.054e+00	-1.142	0.253
		% *Cx.pip.pip*	-1.173e-10	1.491e+00	0.000	1.000
		% Hybrid	2.877e-01	1.394e+00	0.206	0.837
Lower arid	Species	Intercept	-1.09861	0.99999	-1.099	0.272
		% *Cx.pip.pip*	0.09531	1.38169	0.069	0.945
		% Hybrid	-0.10536	1.45295	-0.073	0.942
Saharan	Species	Intercept	-23.30	49252.67	0	1
		% *Cx.pip.pip*	22.89	49252.67	0	1
		% Hybrid	22.22	49252.67	0	1

Description of data

In this study, we collected and identified the *Cx.pipiens* forms from seven bioclimatic regions in Tunisia.

These data represent a study of the relationship between the *Cx. pipiens* forms and the bioclimatic regions, in order to evaluate whether their distribution is depend on climate. We used the Generalized Linear Model (GLM) with Poisson distribution.
