# Supplementary material for: Ecophysiological characterization and molecular differentiation of Culex pipiens forms (Diptera: Culicidae) in Tunisia
Source: Parasit Vectors. 2017 Jul 10;10:327. doi: 10.1186/s13071-017-2265-7 (PMC5504560; doi:10.1186/s13071-017-2265-7)
Supplement: Supplementary file 3 — Relationship between the different types of habitat and breeding site and the proportion of Cx. pipiens based on a Generalized Linear Model (GLM) with Poisson distribution. (PDF 98 kb) [file 13071_2017_2265_MOESM3_ESM.pdf]

Table S3. Relationship between the different types of habitat and breeding site and the proportion of *Cx. pipiens,* based on a Generalized Linear Model (GLM) with Poisson distribution.

Dependent variable	***Independent variable***		***Estimate***	***Standard error***	***Z value***	***P (> |Z|)***
% Cx. molestus	Habitat	Intercept	3.19508	0.06746	47.36	< 2e-16 ***
		Urban	0.31531	0.08277	3.81	0.000139***
% Cx. molestus	Breeding site	Intercept	3.25104	0.04639	70.08	< 2e-16 ***
		Under	0.60969	0.08611	7.08	1.44e-12 ***
% Cx. pipiens	Habitat	Intercept	3.93942	0.04650	84.72	<2e-16 ***
		Urban	-0.85188	0.07531	-11.31	<2e-16 ***
% Cx. pipiens	Breeding site	Intercept	3.6923	0.0372	99.248	<2e-16 ***
		Under	-1.8598	0.2034	-9.142	<2e-16 ***
% Hybrid	Habitat	Intercept	3.18631	0.06776	47.024	< 2e-16 ***
		Urban	0.61177	0.07947	7.698	1.38e-14 ***
% Hybrid	Breeding site	Intercept	3.52765	0.04040	87.326	< 2e-16 ***
		Under	0.30641	0.08389	3.653	0.26

Description of data

The distribution of *Cx. pipiens* forms has been evaluated according to the type of habitat (urban, rural) and the type of breeding site (above and under-ground).

Based on Generalized Linear Model (GLM) with Poisson distribution, these data shows the Relationship between forms and the different types of habitat and breeding site.
