# Supplementary material for: Ecophysiological characterization and molecular differentiation of Culex pipiens forms (Diptera: Culicidae) in Tunisia
Source: Parasit Vectors. 2017 Jul 10;10:327. doi: 10.1186/s13071-017-2265-7 (PMC5504560; doi:10.1186/s13071-017-2265-7)
Supplement: Supplementary file 4 — Autogenic expression in field female mosquitoes. (PDF 168 kb) [file 13071_2017_2265_MOESM4_ESM.pdf]

Table S4. Autogenic expression in field female's mosquito

ID	Locality	Habitat	Breeding site	Number of female	Number of eggs produced	Percentage of autogeny* (%)
**3**	Utique	urban	above	39	5	12.82
**4**	Manar	rural	above	69	1	1.45
**6**	Beja oued	urban	above	29	7	24.18
**9**	Cave 1	urban	under	21	16	76
**11**	Cité olympique	urban	under	37	18	48.64
**13**	Tastour	rural	above	38	0	0

* Number of egg rafts relative to number of female mosquito

Description of data

These data shows an estimated rate of autogeny of *Cx. pipiens* females according to rural and urban habitat, then according to above and under-ground site. Six sites were examined in this test:

- ID3 and ID6 belong to the urban habitat and above-ground site
- ID4 and ID 13 belong to the rural habitat and above-ground site
- ID9 and ID11 belong to the urban habitat and under-ground site
