# Supplementary material for: Ecophysiological characterization and molecular differentiation of Culex pipiens forms (Diptera: Culicidae) in Tunisia
Source: Parasit Vectors. 2017 Jul 10;10:327. doi: 10.1186/s13071-017-2265-7 (PMC5504560; doi:10.1186/s13071-017-2265-7)
Supplement: Supplementary file 5 — Results of the relationship between habitat type and breeding site type and the percentage of autogeny of Cx. pipiens mosquitoes, based on a Generalized Linear Model with Poisson distribution. (PDF 88 kb) [file 13071_2017_2265_MOESM5_ESM.pdf]

Table S5. Results of the relationship between habitat type and breeding site type and the percentage of autogeny of *Cx. pipiens* mosquitos, based on a Generalized Linear Model with Poisson distribution.

**Dependent variable**	**Independent variable**		**Estimate**	**Standard error**	**Z value**	***P* (> |Z|)**
**% *autogeny***	Breeding site	Intercept	2.2620	0.1613	14.02	<2e-16***
		Under	1.8719	0.1845	10.14	<2e-16***
**% *autogeny***	Habitat	Intercept	1.5595	0.2647	5.891	3.83e-09***
		Urban	2.3457	0.2771	8.465	< 2e-16 ***

Description of data

Using a Generalized Linear Model with Poisson distribution, these data shows a statistical analysis of the relationship between the expression of autogeny of the different forms of *Cx. pipiens* and the type of habitat (urban, rural) and the type of breeding site (under-ground, above-ground).
