# Supplementary material for: Ecophysiological characterization and molecular differentiation of Culex pipiens forms (Diptera: Culicidae) in Tunisia
Source: Parasit Vectors. 2017 Jul 10;10:327. doi: 10.1186/s13071-017-2265-7 (PMC5504560; doi:10.1186/s13071-017-2265-7)
Supplement: Supplementary file 6 — Autogeny according to Cx. pipiens form. (PDF 89 kb) [file 13071_2017_2265_MOESM6_ESM.pdf]

Table S6. Autogeny according to *Cx. pipiens* form*

	ID 8 (above)		ID 11 (under)	
	autogenous	anautogenous	autogenous	anautogenous
Cx. p. pipiens	3<br>(13.63)	6<br>(31.58)	1<br>(2.78)	0<br>(0)
Cx. p. molestus	11<br>(50)	5<br>(26.32)	19<br>(52.78)	8<br>(61.54)
**Hybrid**	8<br>(36.36)	8<br>(42.11)	16<br>(44.44)	5<br>(38.46)
**Analyzed female**	22	19	36	13
	41		49	
**Died female**	19		8	

Values in parenthesis refer to the percentage within each form.
*Values in table represent a total of 3 replicates

Description of data

These data show the expression of autogeny according to *Cx. pipiens* form in above and underground sites. This test was replicated three times (once a month). In all, we started with 60 couples from ID8 and 57 from ID11 and at the end of the test; we used molecular analysis only for the survived females (n=41 for ID8; n=49 for ID11). These data represent the total of three replicates.
