# Supplementary material for: Ecophysiological characterization and molecular differentiation of Culex pipiens forms (Diptera: Culicidae) in Tunisia
Source: Parasit Vectors. 2017 Jul 10;10:327. doi: 10.1186/s13071-017-2265-7 (PMC5504560; doi:10.1186/s13071-017-2265-7)
Supplement: Supplementary file 7 — Results of the relationship between percentage of autogeny of Cx. pipiens forms and the type of breeding site, based on a Generalized Linear Model with Poisson distribution. (PDF 313 kb) [file 13071_2017_2265_MOESM7_ESM.pdf]

Table S7. Results of the relationship between percentage of autogeny of *Cx. pipiens* forms and the type of breeding site, based on a Generalized Linear Model with Poisson distribution.

	Dependent variable	Independent variable		Estimate	Standard error	Z value	*P* (> |Z|)
**Above-ground**	*% autogeny*	Species	Intercept	3.9120	0.1414	27.662	< 2e-16***
			Cx pipiens	-1.2997	0.3056	-4.254	2.1e-05***
			Hybrid	-0.3186	0.2180	-1.462	0.144
**Under-ground**	*% autogeny*	Species	Intercept	3.9661	0.1376	28.814	<2e-16***
			Cx pipiens	-2.9437	0.6154	-4.784	1.72e-06***
			Hybrid	-0.1720	0.2036	-0.845	0.398

Description of data

These data show a statistical analysis using a Generalized Linear Model with Poisson distribution, to evaluate the relationship between the percentage of autogeny of *Cx. pipiens* forms and the type of breeding site (under and above-ground).
