# Supplementary material for: Ecophysiological characterization and molecular differentiation of Culex pipiens forms (Diptera: Culicidae) in Tunisia
Source: Parasit Vectors. 2017 Jul 10;10:327. doi: 10.1186/s13071-017-2265-7 (PMC5504560; doi:10.1186/s13071-017-2265-7)
Supplement: Supplementary file 8 — Nucleotide variants in 714 bp of the acetylcholine esterase 2 gene in Cx. pipiens (PDF 160 kb) [file 13071_2017_2265_MOESM8_ESM.pdf]

Table S8. Nucleotide variants in 714 bp of the Acetylcholine esterase 2 gene in *Cx. pipiens*

Accession N°	**Ace 2 gene position** / ***Cx. pipiens* form**	180	267
[AY196910.1]	*Cx. pipiens*	C	G
[JF430595.1]	*Cx. pipiens pipiens*	C	T
[AB294405.1]	*Cx. pipiens molestus*	G	G

Description of data

These data represent the differences in nucleotide sequences of the *Ace 2* gene of three *Cx. pipiens* forms previously described in the world, available in GenBank
